# PPB-Affinity: Protein-Protein Binding Affinity dataset for AI-based protein drug discovery

**DOI:** 10.1038/s41597-024-03997-4

**Published:** 2024-12-03

**Authors:** Huaqing Liu, Peiyi Chen, Xiaochen Zhai, Ku-Geng Huo, Shuxian Zhou, Lanqing Han, Guoxin Fan

**Affiliations:** 1 Artificial Intelligence Innovation Center, Research Institute of Tsinghua, Pearl River Delta, Guangzhou, 510700 China; 2 Cyagen Biosciences (Suzhou) Inc., Guangzhou, 215000 China; 3 Cyagen Biosciences (Guangzhou) Inc., Guangzhou, 510700 China; 4 Cyagen Biomodels (Guangzhou) Co., Ltd, Guangzhou, 510700 China; 5 Department of Pain Medicine, Shenzhen Nanshan People’s Hospital, Shenzhen University Medical School, Shenzhen, 518056 China

**Keywords:** Virtual screening, Drug development

## Abstract

Prediction of protein-protein binding (PPB) affinity plays an important role in large-molecular drug discovery. Deep learning (DL) has been adopted to predict the changes of PPB binding affinities upon mutations, but there was a scarcity of studies predicting the PPB affinity itself. The major reason is the paucity of open-source dataset with PPB affinity data. To address this gap, the current study introduced a large comprehensive PPB affinity (PPB-Affinity) dataset. The PPB-Affinity dataset contains key information such as crystal structures of protein-protein complexes (with or without protein mutation patterns), PPB affinity, receptor protein chain, ligand protein chain, etc. To the best of our knowledge, this is the largest publicly available PPB affinity dataset, and we believe it will significantly advance drug discovery by streamlining the screening of potential large-molecule drugs. We also developed a deep-learning benchmark model with this dataset to predict the PPB affinity, providing a foundational comparison for the research community.

## Background & Summary

Protein-based drugs, including cytokines, enzymes, antibodies, and vaccines, continue to be a major research focus due to their impact on a wide range of diseases including cancer, cardiovascular disease, hepatitis, gastrointestinal disease, autoimmune disease, and transplant rejection. However, there are still many challenges in the development of protein drugs, one of which is the screening efficiency of drug candidates. The prediction of protein-protein binding (PPB) affinity is a crucial step in the screening process of protein drugs. Protein drugs usually exert their effects by binding to specific receptors, frequently other proteins. Thus, a higher PPB affinity translates to stronger binding between the drug and the receptor, which in many cases may lead to improved therapeutic efficacy. Prior studies have primarily focused on predicting the changes of PPB binding affinity upon mutations^1–9^, which only allows the discovery of structure-similar protein drugs. The datasets for such studies typically include SKEMPI v2.0 dataset^10^, AB-Bind^11^, and some deep mutagenesis datasets^12–18^. Only a few studies endeavored to predict PPB affinity, most of which focused in predicting antigen-antibody binding affinity or TCR-pMHC binding affinity^19–26^, but the prediction accuracy has fallen short of practical application.

The difficulty of predicting PPB affinity lies in the development of prediction algorithms and the sources of data, and data scarcity hinders algorithm development. Currently available datasets include SKEMPI v2.0, AB-Bind, SAbDab^27–29^, PDBbind v2020 (http://pdbbind.org.cn/)^30–35^, Affinity Benchmark v5.5^25,26,36^, and ATLAS^37^. The SKEMPI v2.0 dataset contains 7085 samples of affinity changes upon mutations in protein-protein complexes, including data such as crystal structures of wild-type complexes, protein mutation patterns, and the magnitude of affinity changes. However, the SKEMPI v2.0 dataset only included 345 crystal structures, because most of the samples are mutant type. The AB-Bind dataset is included within the SKEMPI v2.0 dataset. The SAbDab dataset is a collection of antibody crystal structure data, containing over 7000 antibody-antigen binding crystal structures, but only a small portion of these samples records the PPB affinities. The PDBbind v2020 dataset contains 23,496 crystal structures of biomolecular complexes and their affinity data, covering protein-small molecule ligand complexes, protein-protein complexes, protein-nucleic acid complexes, and nucleic acid-small molecule complexes. There are 2789 samples of protein-protein complexes in the PDBbind v2020 dataset, but it does not explicitly indicate the receptor protein chain and ligand protein chain in the complexes. The Affinity Benchmark v5.5 dataset provides crystal structures of protein-protein complexes and their affinities, but it only consists of 207 protein-protein samples. ATLAS provides affinity data for TCR and its antigen (peptide-MHC complex) binding, affinity changes upon mutations, and complex crystal structures, but it has only 694 samples, with only 112 complex crystal structures obtained from biological experiments. Additionally, the above public databases might fail to include some PPB affinity data promptly, which were experimentally measured and published by most recent studies^38–40^. In summary, there is a paucity of comprehensive public datasets with experimentally measured information for the AI prediction of PPB affinity.

The quality and sample size of experimental data, as well as the diversity of samples, are essential to achieve accurate and highly generalized PPB prediction. Although machine learning (ML) algorithm, especially deep learning (DL) technique has been validated in optimizing multiple steps of drug discovery, there is a paucity of ML or DL studies predicting PPB affinity. To address this gap, we introduce the PPB-Affinity dataset, the largest publicly available comprehensive dataset meticulously integrated and processed from all currently available public data with protein-protein complex crystal structures and their affinities. We believe PPB-Affinity will significantly enhance drug discovery efficiency in the pharmaceutical industry by streamlining the screening of potential large-molecule drugs.

## Methods

This study has been exempted by the local ethics committee because the original data are extracted from the publicly available online datasets and then semi-automatic processed.

### Data processing and quality assessment

**Fig. 1:**
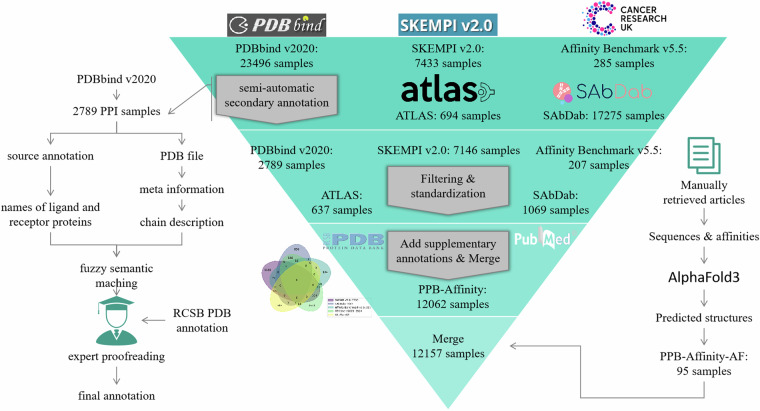
Schematic workflow of creating the PPB-Affinity database.

We integrated and processed multiple related open-source datasets through a combination of automatic processing and manual verification (Fig. 1). The source datasets utilized in this study were SKEMPI v2.0, SAbDab (by July 25^th^, 2024), PDBbind v2020, Affinity Benchmark v5.5, and ATLAS. The crucial information for PPB-Affinity dataset includes the experimentally measured affinity values, the crystal structures of protein-protein complexes (particularly the crystal structures of the binding interfaces), ligand protein chains, and receptor protein chains. To be noted, many samples involve mutant protein complexes, where only the crystal structures of their corresponding wild-type complexes are available. For these samples, we also provide information on the mutation patterns. Additional supplementary information includes the source dataset, experimental temperature for affinity measurement, affinity measurement methods, crystal structure determination methods, and relevant references.

#### Affinity

Different source datasets provide affinity information in various formats. For instance, SKEMPI v2.0 provides dissociation constant (KD) values in molar units (M), while some samples from SAbDab present both KD values and change in Gibbs free energy upon binding (∆G) values. PDBbind v2020, on the other hand, offers KD, inhibition constant (Ki), or half maximal inhibitory concentration (IC50) values, with units ranging from millimolar (mM), micromolar (uM), to nanomolar (nM). ATLAS, meanwhile, provides KD values in mM units. To ensure consistency, we have standardized the affinity representation across all samples, expressing them uniformly as KD values in molar units (M). Notably, IC50 values cannot be directly converted to KD values, and we have excluded them from our dataset as these samples constitute a minority. This approach allows for a more coherent and comparable analysis of PPB affinity across different datasets.

#### Ligand and receptor types

We only included samples from the source dataset where both receptors and ligands are proteins or polypeptides.

#### Protein chains

The affinity of PPB physically refers to the change in free energy before and after the binding of receptor proteins and ligand proteins. The greater the reduction in free energy after binding, the stronger the affinity. Therefore, computational methods, including ML, must distinguish between receptor proteins and ligand proteins. Our dataset annotates the ligand protein chains and receptor protein chains in each protein-protein complex. Some source datasets, such as SKEMPI v2.0, ATLAS, and Affinity Benchmark v5.5, have already annotated ligand protein chains and receptor protein chains. For these source datasets, we directly incorporate their annotations and correct a few mislabeled ones. For example, in the Affinity Benchmark v5.5 dataset, the ligand and receptor labeled for 3A4S are chains A and D, respectively. However, there is no binding interface between these two chains in PDB. The correct annotation should be chains B and C. For 4FQI in the Affinity Benchmark v5.5 dataset, the originally labeled receptor and ligand chains are H, L and A, B, E, F, C, D, respectively. Nevertheless, chains E, F, and D do not exist in the PDB. Although chain C exists, it is not an amino acid chain and is far from the binding interface, so it is also excluded. The corrected annotation is chains H and L as ligands, and chains A and B as receptors. Although the protein-protein subset of PDBbind v2020 annotates the names of ligand proteins and receptor proteins in the complex, it does not clearly annotate the chains of ligand proteins and receptor proteins. We used the following semi-automatic annotation method for these samples:

1. For each sample, read the text names of ligand proteins and receptor proteins annotated by PDBbind v2020;
2. Read the meta-information of the corresponding PDB file for the sample and obtain the descriptive text for each protein chain;
3. Use a fuzzy semantic matching method to calculate the matching degree between the ligand and receptor protein name texts and any protein chain description text;
4. Based on the matching degree, identify the most likely ligand protein chain and receptor protein chain;
5. Expert proofreading: Structural biology experts observe the crystal structure and sequence of the PDB file, combined with annotation information from the RCSB Protein Data Bank (RCSB PDB) to manually proofread the protein chains found through semantic matching;
6. Splitting: A PDB file may contain multiple identical ligand-receptor complexes. Structural biology experts observe the crystal structure and sequence in the PDB file, referring to information from the RCSB PDB database, to split and annotate each independent complex. Through this method, we annotated ligand protein chains and receptor protein chain information for 2788 samples.

It is noted that the SAdDab database annotates antigen chains, antibody heavy chains, and antibody light chains. For these samples, we annotate the antigen chain as the receptor chains and the antibody heavy and light chains as the ligand chains. The ATLAS dataset includes affinity data for TCR and p-MHC binding and annotates limited information such as mutated TCR chains and peptide sequences. We identify TCR, peptide-MHC molecules in PDB files through structural biology expert observation, and annotate TCR as the ligand chain and peptide-MHC as the receptor chain. To trace the original data, we also annotate the dataset from which each sample originates and record the original reference as completely as possible. Some records exist in two or more different source datasets, and when merging the source datasets, we only kept one of these records and deleted the rest of the duplicates. To address the cross-source consistency issue, we assign a unique Complex ID for each record in the source dataset, defined by the following formula using the PDB code, ligand and receptor chain codes (sorted), mutation information (sorted), and PubMed ID (the reference for measuring the affinity):

Complex ID = f“{pdb}:{chains}:{mutations}:PMID = {affinity_PMID}”

Samples with the same Complex ID were considered the same sample, which means that their structures and sequences are consistent, and the affinity measurement values come from the same experiment. Therefore, when merging different source datasets based on the Complex ID, we only keep one of the samples with the same Complex ID. In this way, we merged the 5 source datasets as the PPB-Affinity dataset. In other words, there are a few samples that have the same PDB code, ligand, and receptor chain codes (sorted), and mutations, but they have different PubMed IDs, indicating that they come from different references, and the conditions of the affinity measurement experiments may vary, as may the affinity measurement values. As a result, the PPB-Affinity dataset regarded these samples as different samples, and annotated the affinity measurement experimental conditions (including the measurement method, environmental temperature, pH value, etc.) and the size of the affinity for each sample. Additionally, some unqualified data were deleted during manual screening, and the deletion details of samples are listed as follows:

1. Lack of annotated affinity: There are 57 records without annotated affinity in the SKEMPIv2.0 dataset; the Affinity Benchmark v5.5 has 78 records without annotated affinity; the SAbDab dataset has 14,148 records without annotated affinity.
2. Records that are not protein-protein complexes: 6 non-protein-protein complexes in the PDBbind v2020 dataset and 46 such records in the SAbDab dataset.
3. Errors or inability to annotate protein chains: We used a semi-automatic method plus expert review to annotate ligand chain IDs and receptor chain IDs for each record in the PDBbind v.2020 dataset, and for 62 samples, we could not clearly annotate them, so they were not included in our dataset. In addition, 95 records in SAbDab have no antigen chain (receptor chain) annotation; there are 8 records with chain annotation errors because the annotated (antibody heavy chain, light chain, or antigen chain) does not exist in the corresponding PDB file.
4. The annotated affinity cannot be converted into KD values: There are 62 such samples in PDBbind v2020. Specifically, their affinities are not KD values, Ki values, or ΔG values, but are IC50/EC50 values, etc. Although IC50 or EC50 can be mathematically converted into KD values, the conversion cannot be completed due to the lack of some necessary variable information in the formula (such as substrate concentration, etc.).

We also created a sub-dataset called “PPB-Affinity-AF”, which were collected from most recent studies publishing the experimentally measured affinity, and their three-dimensional structure of the complex were created with AphaFold3. The including criteria of the collected samples were: 1. The ∆G or KD of the protein has been determined through experiments, 2. The protein sequence has been published, but there is no crystal structure of the protein complex determined by instruments. These data are scattered in different papers, so we tried our best to collect them from different papers that we could identify. Then we manually organized the protein sequence, affinity, and other information for each sample. A total of 95 samples were finally included, and we used AlphaFold3 to predict the corresponding three-dimensional structure of the complex and saved them as PDB files.

## Data Record

The PPB-Affinity dataset^41^ is available on https://zenodo.org/doi/10.5281/zenodo.11070823. The dataset is now accessible under the Creative Commons Attribution 4.0 International, which supports its use for educational and research purposes. Users should cite this paper when they incorporate the dataset into their projects. The presented dataset consists of two parts. The first part is an excel spreadsheet (summary.xlsx) that summarizes the annotation information of all samples in the dataset. The second part is a folder containing the crystal structure (PDB files) of all samples in the dataset.

### Data format

1. PDB: pdb code of the crystal structure of the protein-protein complex
2. Source dataset: refer to the sample source of the original datasets
3. Model: refer to which model in the PDB that the target complex belongs to, the default is 0
4. Mutations: refer to the mutations of ligand proteins and receptor proteins and the format is [PDB code]_[reference animo acid][mutation site][alteration animo acid]
5. Ligand Chains: refer to the ligand protein chains
6. Recaptor Chains: refer to the receptor protein chains
7. Ligand Name: refer to the receptor protein name
8. Receptor Name: refer to the receptor protein name
9. KD(M): the KD value of affinity and the unit is M
10. ∆G(kcal/mol): The change difference of the Gibbs free energy when the receptor and the ligand binds together, which is transformed from the KD value (Environment temperature is 25°C, and the unit is kcal/mol).
11. Affinity Method: measurement methods of affinity
12. Structure Method: measurement methods of crystal structures
13. Temperature(K): experiment temperature when measuring the affinity, and the unit is K
14. Resolution(Å): resolution of the crystal structure, and the unit is Å
15. PDB PubMed ID: PubMed ID of the literature that disclose the crystal structure
16. PDB Release Date: release date of the crystal structure
17. Affinity PubMed ID: PubMed ID of the literature that disclose the affinity of the proteins

### Metadata

**Fig. 2:**
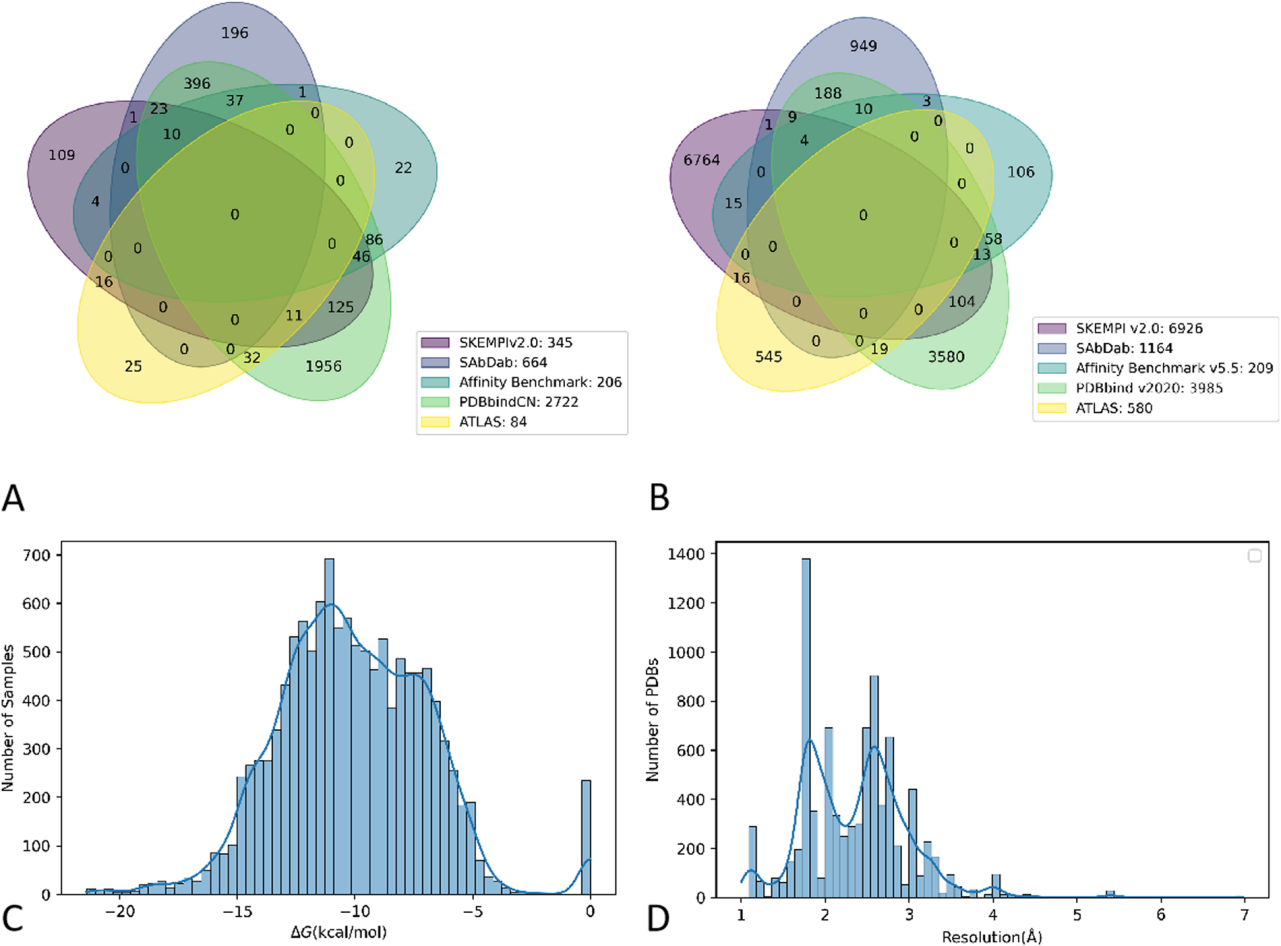
Distribution summary of the whole dataset. (**A**) unique PDB codes distributed among different source datasets; (**B**) unique samples distributed among different source datasets; (**C**) sample affinity of the whole dataset; (**D**) PDB resolution of the whole dataset.

**Fig. 3:**
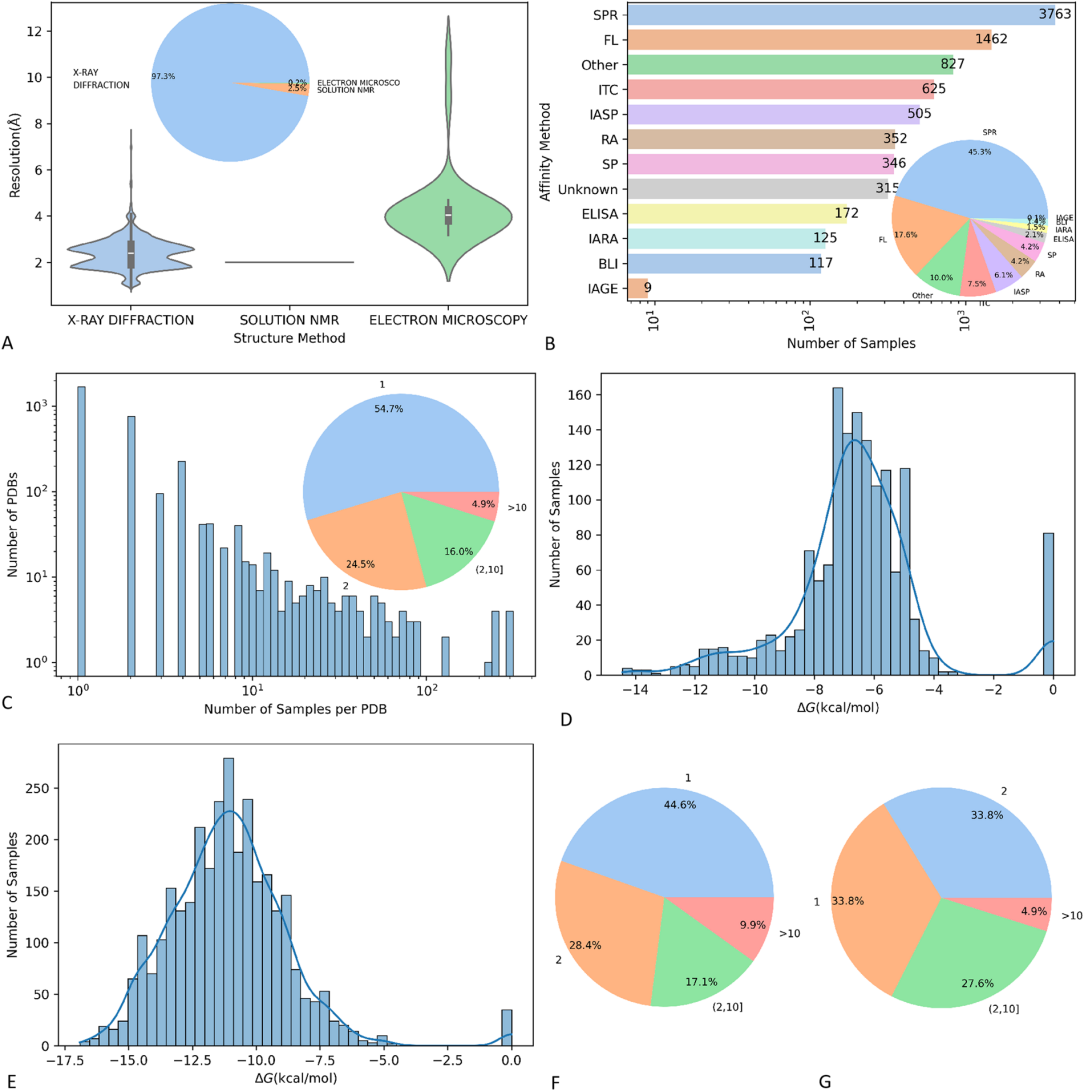
Meta-data information of the entire dataset and subgroup distribution. (**A**) Summary of structural determination methods; (**B**) Summary of affinity determination methods.; (**C**) distribution of mutant patterns for the entire dataset. (**D**) affinity distribution of TCR-pMHC subgroup; (**E**) affinity distribution of antibody-antigen subgroup; (**F**) mutant patterns of TCR-pMHC subgroup; (**G**) mutant patterns of antibody-antigen subgroup.

We identified a total of 3032 unique PDB codes and the intersection situation among different source datasets was presented with Venn diagrams (Fig. 2A). We also identified a total of 12062 unique samples, which referred to unique PDB, ligand chains, receptor chains, and mutations (Fig. 2B). We used histograms to present the PDB resolution (Fig. 2C) and the distribution of sample affinity (Fig. 2D) of the whole dataset. We summarized the proportion of different structural determination methods for all samples in the dataset and the distribution of structural resolution by different structural determination methods (Fig. 3A). Of the 306 samples with crystal structures determined using solution NMR, only two of them had recorded resolution values. The average resolution of the structures determined by X-RAY diffraction was slightly higher than that of electron microscopy. Additionally, the number of different affinity determination methods for each sample was also presented. As Fig. 3B presented, surface plasmon resonance (SPR) was the main method (43.6%) of determining affinity in samples with known affinity measurement methods. We also summarized the distribution of mutant patterns for the whole dataset. As Fig. 3C presented, 54.6% of PDBs associated with single samples (wild-type), 24.3% associated with double samples (1 wild-type and 1 mutant-type), 16.4% associated with multiple samples (1 wild-type and 1–9 mutant-type), and 4.7% associated with large number of samples (1 wild-type and more than 10 mutant-type). As shown in Fig. 3D,E, the affinity of TCR-pMHC binding was primarily concentrated between −8 to −5.5 kcal/mol, which was weaker than the affinities observed in antigen-antibody interactions and other common protein interactions. This phenomenon could be explained by the monitor and regulate role of TCRs in the immune system. As illustrated in Fig. 3F,G, both the antibody-antigen subgroup and the TCR-pMHC subgroup contained a higher proportion of mutant samples compared to the entire dataset.

### Data diversity

In the analysis of protein-protein interactions, sequence alignment is often performed on complexes to determine homology/similarity. Homologous proteins are defined as those with a similarity score greater than 50% and at least 30% sequence identity. Given that affinity is highly correlated with the three-dimensional structure of the binding interface, we adopted a novel method called IDist^9^ to present the similarity network of binding interface of all included complex. In this method, binding sites are defined as locations where the Euclidean distance between any CA atom in the receptor protein and any CA atom in the ligand protein is less than 10 Å. The SE(3) feature vectors of these binding sites are then calculated using the IDist algorithm and then the representation of the binding interfaces were obtained by integrating SE(3) features of all binding spots. If the Euclidean distance between the representations of any two binding interfaces is less than 0.05, these interfaces are defined as similar, and in the graph, this similarity is represented by connecting the corresponding nodes.

**Fig. 4:**
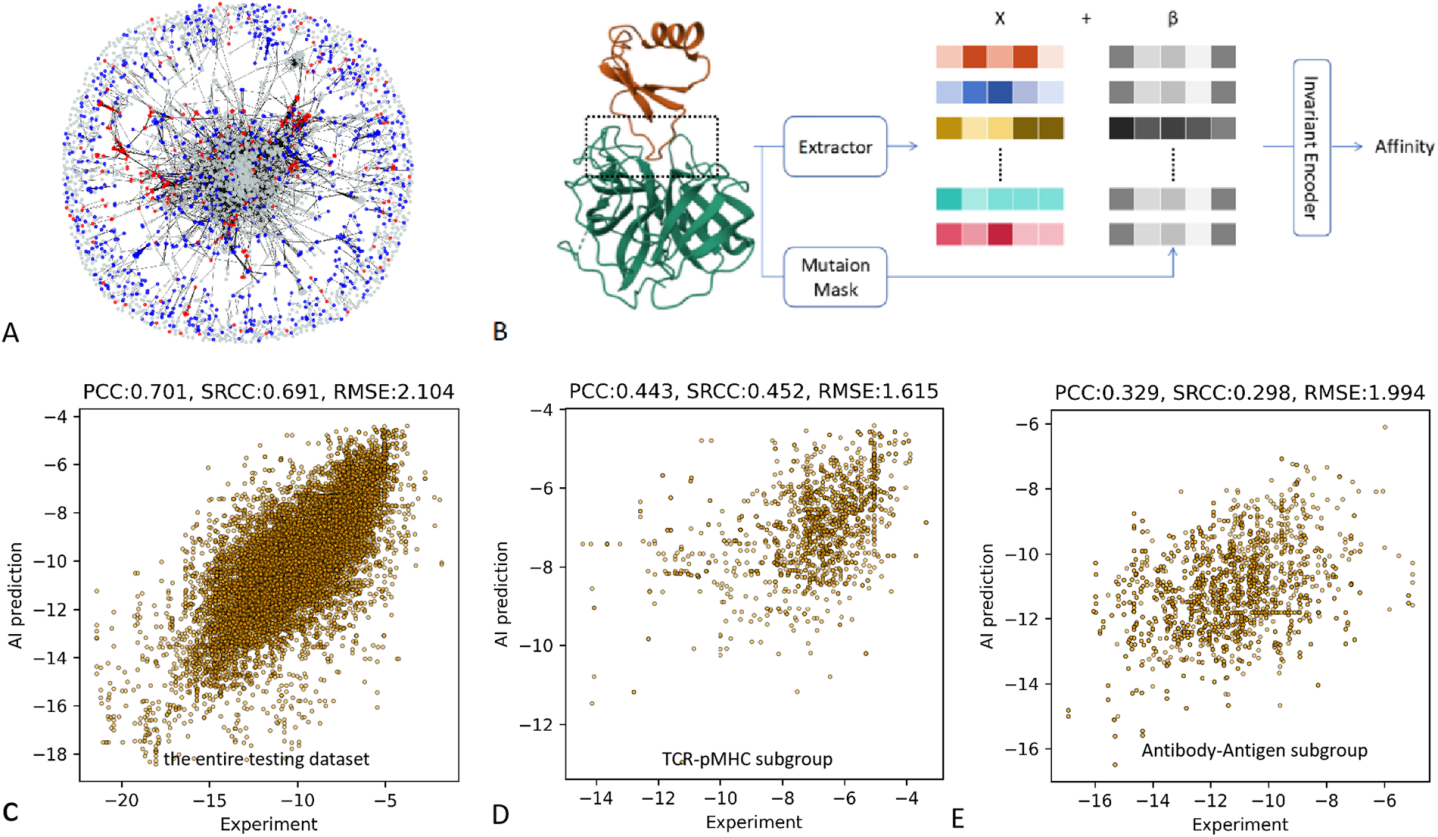
Visualization of data diversity and benchmark affinity prediction. (**A**) similarity network of binding interface, while blue nodes representing antibody-antigen complexes, red nodes representing TCR-pMHC complexes, and gray nodes representing other proteins; (**B**) design of benchmark algorithm for affinity prediction; (**C**) performance of the benchmark model for the entire testing dataset; (**D**) performance of the benchmark model for the TCR-pMHC subgroup; (**E**) performance of the benchmark model for the antigen-antibody subgroup.

In Fig. 4A, each node represents a complex, with blue nodes representing antibody-antigen complexes, red nodes representing TCR-pMHC complexes, and gray nodes representing other proteins. Edges indicate that the two connected nodes have similar protein-protein binding interfaces. It was noted that the TCR-pMHC subgroup exhibits a high degree of clustering. This is likely due to the high similarity in the binding interfaces of diverse TCR-pMHC complexes. On the other hand, antibody-antigen complexes also showed sort of clustering, but the degree of aggregation was significantly lower than that of the TCR-pMHC subgroup. This could be explained by the fact that although the variable region of antibodies had a certain degree of conserved structure, its diversity was higher than that of TCRs. More importantly, antigens exhibit a wide range of sequences and structures, leading to a higher diversity in the binding interfaces of antibody-antigen complexes.

## Technical Validation

### A benchmark affinity prediction

We proposed a benchmark algorithm based on geometric deep learning method (Fig. 4B). The core idea is to use the IPA (invariant point attention)^42^ method to extract features from the crystal structure of protein-protein complexes to predict the affinity magnitude. Firstly, 128 amino acid residues at the ligand-acceptor binding interface and its immediate neighbors were intercepted. The amino acid residue sequence was input to the residue encoder for residue encoding. The extractor was designed to extract features from both individual amino acids and pairs of amino acids. Specifically, the type, position, dihedral angle and other information of amino acids serve as note features, while the type, relative position, distance, virtual dihedral angle and other information of amino acid pairs constitute edge features. These features were collectively referred to as residue feature X, since some amino acids in X may undergo mutated. To account for these mutations, a mutation mask was introduced to indicate any animo acid alterations. This mask was then embedded into a vector, denoted asβ, and incorporated to X. Subsequently, the residue feature X and its 3-dimensional spatial coordinates were fed into the spatially invariant point attention module. This module was expected to extract the rotation and translational invariant spatial features of the residues. Finally, the spatial characteristics of the residues were integrated to predict the affinity magnitude through the prediction head.

### Whole dataset

As shown in Fig. 4C, the Benchmark algorithm achieved a Pearson correlation coefficient of 0.701 and a Spearman correlation coefficient of 0.691 on this dataset. However, there were some weak horizontal lines visible in the chart. This was because the benchmark model might have difficulty capturing the differences between certain mutants and their wild types, hence it predicted similar or identical affinity values. The main reasons are: 1) the dataset does not provide the crystal structures of the mutant-type samples, and 2) the benchmark algorithm itself is weak to capture the characteristics of the mutant-type crystal structures based on the wild-type crystal structures and their mutation information.

### Antibody-antigen subgroup

In the antibody-antigen subgroup, the correlation between the predicted values by the benchmark model and the actual values was significantly lower than that of the entire dataset (Fig. 4D). The main reasons for this discrepancy are likely as follows: 1) One of the reasons for this discrepancy may be due to the high structural similarity of antibodies, making it difficult for current technologies or algorithms to distinguish such structurally similar proteins; 2) Another reason could be the diversity and uncertainty of antigens and antigenic epitopes; 3) compared to the overall dataset, there is a higher proportion of mutant samples in the antibody-antigen subgroup, whose complex crystal structures are unknown. The benchmark algorithm lacks the capability to capture the characteristics of mutated structures.

### TCR-pMHC subgroup

Similarly, the correlation between the predicted values and the actual values for the TCR-pMHC subgroup by the benchmark model was lower than that of the overall dataset, and slightly higher than that of the antibody-antigen subgroup (Fig. 4E). The reasons may be attributed to the fact that not only the structures of TCR is highly similar, but also the peptide-MHC complex looks alike.

### Source datasets

**Fig. 5:**
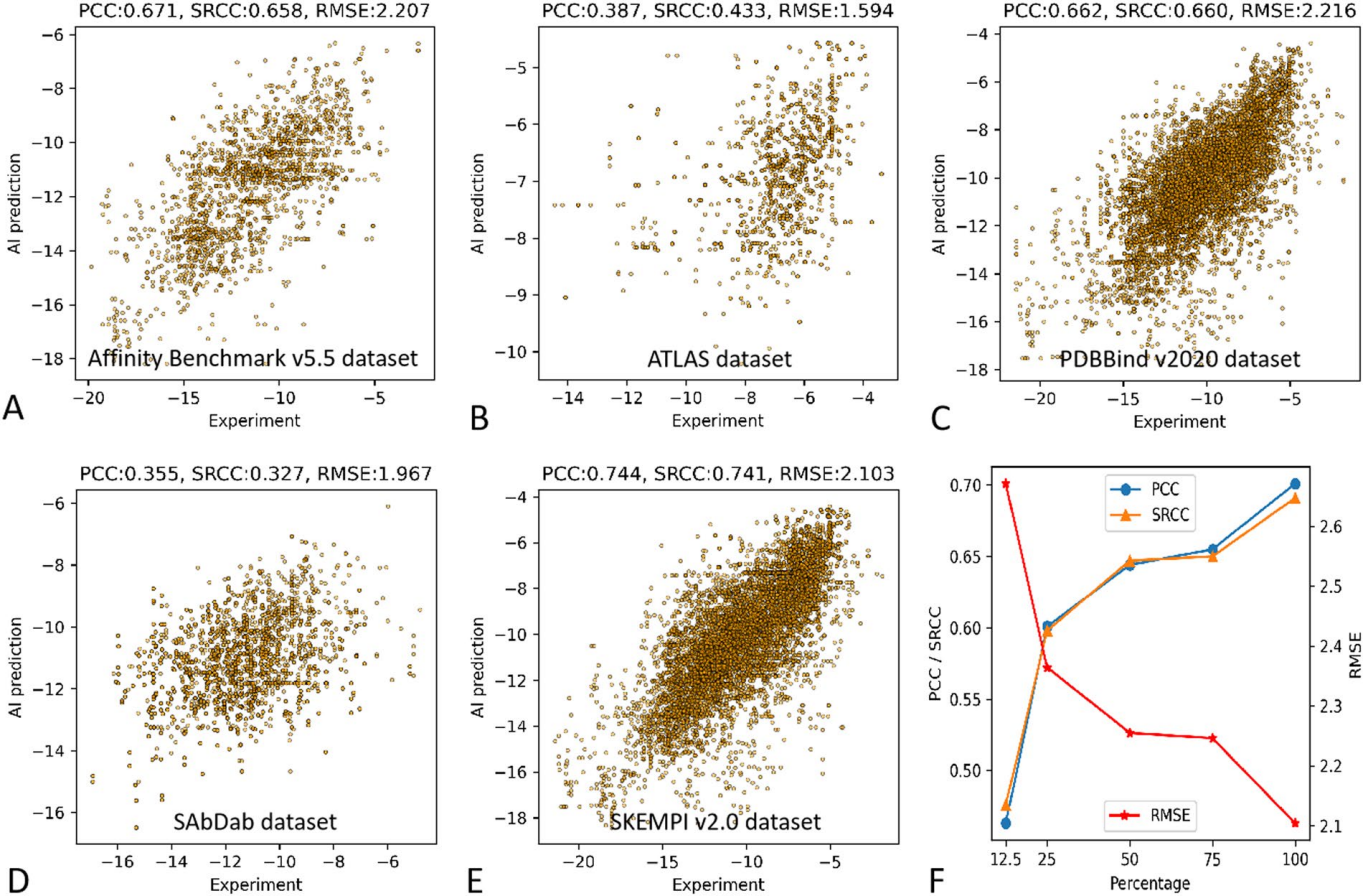
Benchmark affinity prediction for the source datasets; (**A**) performance of the benchmark model for the Affinity Benchmark v5.5 dataset; (**B**) performance of the benchmark model for the ATLAS dataset; (**C**) performance of the benchmark model for the PDBBind v2020 dataset; (**D**) performance of the benchmark model for SAbDab dataset; (**E**) performance of the benchmark model for the SKEMPI v2.0 dataset; (**F**) improved prediction performance with more training data.

We also demonstrated the cross-dataset performance of the benchmark affinity prediction. It seemed that the testing samples from the Affinity Benchmark v5.5 dataset, the PDBBind v2020 dataset and the SKEMPI v2.0 dataset showed higher accuracy than the other two source datasets (Fig. 5A–E). Additionally, we trained the same algorithm with various percentage of the entire dataset to demonstrate the advantages of the current dataset. Results demonstrated that same algorithm trained with more and more training data did showed improved prediction performance (Fig. 5F).

### PPB-Affinity-AF subdataset

**Fig. 6:**
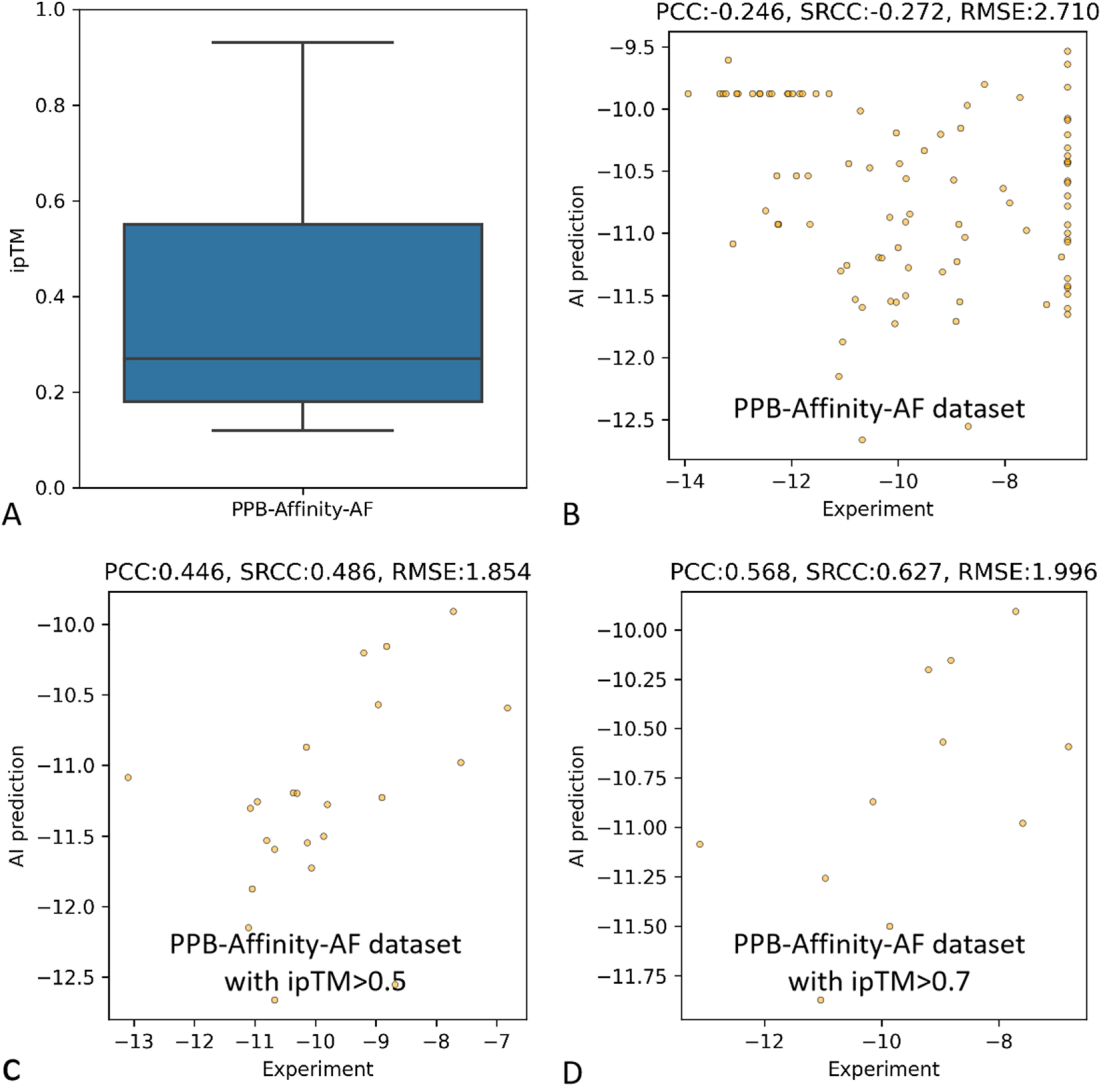
Benchmark affinity prediction for the PPB-Affinity-AF subdataset. (**A**) distribution of the interface predicted template modeling (ipTM) of the complex from the PPB-Affinity-AF subdataset; (**B**) affinity prediction results of all samples from the PPB-Affinity-AF subdataset; (**C**) affinity prediction results of samples with ipTM over 0.5; (**D**) affinity prediction results of samples with ipTM over 0.75.

As the complex structure were not experimentally measured, we demonstrated distribution of the interface predicted template modeling (ipTM) of the complex from the PPB-Affinity-AF subdataset (Fig. 6A), which indicated the prediction reliability of the complex. Although the affinity prediction of the entire PPB-Affinity-AF subdataset seemed to be unsatisfied (Fig. 6B), the affinity prediction accuracy got further improved when the ipTM of samples were from 0.5 to 0.75 (Fig. 6C,D), which further indicate the reliability of the benchmark algorithm and the importance of complex structures.

## Usage Notes

Instructions for use supplement, including:

The dataset can be publicly accessed when you agree to cite the DOI of the collection for your publication resulting from this dataset.

### Potential uses of the dataset

We hope that this dataset will help researchers, and AI algorithm engineers in the field of protein drug discovery to develop more powerful protein-protein binding affinity prediction algorithms.

### Limitations of datasets

First, all samples are currently from other publicly available datasets. Thus, new research papers may sporadically publish experimentally measured protein-protein binding affinity with their crystal structures, but these data might not be promptly collected into the PPB-Affinity dataset. Second, it should be noted that many mutant protein complexes did not have their experimentally measured crystal structures, where only the crystal structures of their corresponding wild-type complexes are available. Third, we only provided the benchmark prediction performance with one mainstream algorithm. As a dataset article, we hope this dataset can attract more researchers to develop more creative algorithms to promote drug discovery in this field.

The meaning of the dataset and the future work of our team: The unique features of our dataset could be summarized as follows: (1) all of the samples have unified affinity indicator, recorded experimental condition and annotated ligand chain and receptor chain, which are not always available for all the source datasets; (2) we also have a sub-dataset called “PPB-Affinity-AF”, which were collected from most recent studies publishing the experimentally measured affinity, and their three-dimensional structure of the complex were created with AphaFold3. The future work will include and develop more experimental data and crystal structure of PPB affinity.

## Data Availability

Codes for PPB-Affinity database preparation is disclosed at https://github.com/Huatsing-Lau/PPB-Affinity-DataPrepWorkflow. Codes for benchmark algorithm is disclosed at https://github.com/ChenPy00/PPB-Affinity.
